# Host obesity alters the ovarian tumor immune microenvironment and impacts response to standard of care chemotherapy

**DOI:** 10.1186/s13046-023-02740-y

**Published:** 2023-07-12

**Authors:** Yueying Liu, Jing Yang, Tyvette S. Hilliard, Zhikun Wang, Jeff Johnson, Wanrui Wang, Elizabeth I. Harper, Connor Ott, Caitlin O’Brien, Leigh Campbell, Brian Crowley, Stephen Grisoli, Nicholas M. Stavrou, Anna Juncker-Jensen, M. Sharon Stack

**Affiliations:** 1 Department of Chemistry & Biochemistry, University of Notre Dame, Notre Dame, IN USA; 2 Harper Cancer Research Institute, University of Notre Dame, A200E Harper Hall, 1234 N. Notre Dame Ave, South Bend, IN 46617 USA; 3 NeoGenomics Laboratories, Aliso Viejo, CA USA

**Keywords:** Ovarian cancer, Obesity, High fat diet, Tumor-associated macrophage, Fibrosis

## Abstract

**Background:**

The majority of women with epithelial ovarian cancer (OvCa) are diagnosed with metastatic disease, resulting in a poor 5-year survival of 31%. Obesity is a recognized non-infectious pandemic that increases OvCa incidence, enhances metastatic success and reduces survival. We have previously demonstrated a link between obesity and OvCa metastatic success in a diet-induced obesity mouse model wherein a significantly enhanced tumor burden was associated with a decreased M1/M2 tumor-associated macrophage ratio (*Liu Y *et al*. Can, Res. 2015; 75:5046–57*).

**Methods:**

The objective of this study was to use pre-clinical murine models of diet-induced obesity to evaluate the effect of a high fat diet (HFD) on response to standard of care chemotherapy and to assess obesity-associated changes in the tumor microenvironment. Archived tumor tissues from ovarian cancer patients of defined body mass index (BMI) were also evaluated using multiplexed immunofluorescence analysis of immune markers.

**Results:**

We observed a significantly diminished response to standard of care paclitaxel/carboplatin chemotherapy in HFD mice relative to low fat diet (LFD) controls. A corresponding decrease in the M1/M2 macrophage ratio and enhanced tumor fibrosis were observed both in murine DIO studies and in human tumors from women with BMI > 30.

**Conclusions:**

Our data suggest that the reported negative impact of obesity on OvCa patient survival may be due in part to the effect of the altered M1/M2 tumor-associated macrophage ratio and enhanced fibrosis on chemosensitivity. These data demonstrate a contribution of host obesity to ovarian tumor progression and therapeutic response and support future combination strategies targeting macrophage polarization and/or fibrosis in the obese host.

**Supplementary Information:**

The online version contains supplementary material available at 10.1186/s13046-023-02740-y.

## Background

Ovarian cancer (OvCa) is the third most common gynecologic cancer globally with 313,959 new diagnoses and 207,252 deaths reported in 2020 [1]. The age-standardized incidence rate is 6.6 per 100,000, representing a decreasing trend in global incidence, but with a substantial increase in reported incidence in younger women aged 15–40 [1]. The majority of women with OvCa are diagnosed with metastatic disease, resulting in a poor 5-year survival of 31% due to painful complications resulting from widely disseminated intra-peritoneal (i.p.) metastases. Metastasis occurs from direct extension of the primary ovarian or fallopian tube tumors and exfoliation of single cells and multicellular aggregates (spheroids or tumorspheres) into the peritoneal cavity, wherein dissemination is facilitated by the accumulation of ascites fluid [2, 3]. Metastatic cells and multicellular aggregates survive in ascites, adhere to the mesothelial cells of the peritoneal membrane that covers abdominal organs, induce mesothelial cell retraction, anchor in the collagen-rich sub-mesothelial extracellular matrix, and proliferate to form widely disseminated secondary lesions [2–5].

Obesity is a recognized noninfectious pandemic [6] that increases OvCa incidence, enhances metastatic success and reduces survival [7–14]. Meta-analyses, including a new study evaluating 2.7 million women [15], show a relationship between obesity and risk of OvCa incidence in women with tumors of serous, endometrioid, and mucinous histology. Importantly, obesity has an adverse effect on survival of women with OvCa, implicating a link between host obesity, metastatic success, and response to therapy [6–16]. This survival effect is highlighted by a recent study that evaluated genomic and clinical data from a cohort of > 600 women diagnosed with high grade serous OvCa (HGSOC) having similar mutational profiles. The authors identified a “poor outcome” cohort associated with upregulated obesity- and lipid metabolism-related genes who have significantly reduced progression-free and overall survival relative to women with the same mutational profile but lacking this gene cluster [17].

These epidemiologic data are consistent with pre-clinical data showing preferential homing of metastasizing ovarian tumors to the omental fat pad [2, 3]. Data examining ex vivo human tumor-adipocyte interactions showed that adipocytes promote homing, migration, and invasion of OvCa cells and transfer fatty acids to promote tumor cell growth [18]. These in vitro data are consistent with the increased tumor aggressiveness observed in a genetically engineered murine OvCa model when comparing mice on a low fat diet (LFD) to those on a high fat diet (HFD), showing increased tumor size and associated metabolic changes [19]. We have previously demonstrated a positive link between obesity and OvCa metastatic success in multiple murine pre-clinical models of diet induced obesity and mutational obesity [20]. Obesity was found to significantly enhance i.p. metastatic tumor burden and lipid transport into tumor cells and was associated with changes in the immune microenvironment reflected by a decreased M1/M2 macrophage ratio [20]. The objective of the current study was to evaluate response to standard-of-care chemotherapy in pre-clinical models of diet induced obesity and to assess obesity-associated changes in the tumor immune landscape in human and murine tumors. Our results show a significantly diminished response to chemotherapy in pre-clinical studies using mice on a HFD. A corresponding decrease in M1/M2 macrophage ratio and enhanced fibrosis in both murine and human tumors suggests potential mechanisms by which host obesity contributes to poor outcomes in women with ovarian cancer.

## Methods

### Murine diet-induced obesity and ovarian cancer allograft model

Cohorts of female C57Bl/6 (age 3–6 weeks, Jackson Labs) were randomly assigned to a low fat diet (LFD, 10% fat, Research Diets #D12450J) or high fat diet (HFD, 45% fat, Research Diets #D12451) cohort and weighed weekly until a ~ 10 g difference in average cohort weight was obtained (~ 11 weeks). Mice were maintained on the assigned diet throughout the study. Allograft studies used the murine C57Bl/6 syngeneic ovarian cancer cell line ID8Trp53^−/−^, generously provided by Dr. McNeish, Glasgow, UK [21], and tagged with red fluorescent protein (RFP) as previously described [22]. ID8Trp53^−/−^ cells were cultured in Dulbecco’s Modified Eagle Medium (Corning) containing 4% Fetal Bovine Serum (Gibco), 1% Penicillin/Streptomycin (Sigma), and 1% Insulin–transferrin–sodium selenite (Sigma). ID8Trp53^−/−^-RFP-tagged cells (1 × 10^7^ in 1 ml PBS) were injected intra-peritoneally (i.p.) and mice were imaged weekly under isoflurane anesthesia using the Ivis Lumina in vivo imaging system. When longitudinal images showed equivalent tumor burden (~ 3 weeks), mice were treated with weight-adjusted standard-of-care chemotherapy [paclitaxel (6 mg/kg) & carboplatin (15 mg/kg) for 6 or 9 cycles, as indicated. Paclitaxel and carboplatin were purchased from Sigma-Aldrich, St. Louis, MO. Throughout the study, mice were observed for signs of lethargy and ascites accumulation. The mice were euthanized for end-point dissection at one week following the last chemotherapy treatment.

Following euthanasia, ascites was collected using a syringe. The ventral skin was then pulled away and the peritoneal cavity was exposed with a midline incision and incisions at the sides of the ventral parietal peritoneum. Abdominal tumor burden was examined as previously described by scanning abdominal organs in situ using the Ivis Lumina in vivo Imaging system, followed by removal of individual organs and ex vivo imaging [22–24]. Total abdominal or organ-specific tumor burden was analyzed using ImageJ by calculating the tumor area and the intensity of the RFP signal (Raw Integrated Density). Statistical analysis was performed using Student’s *t*-test (Sigmaplot) with a *p*-value cutoff of 0.05 defined as statistically significant. All animal procedures were carried out according to the regulations of and with approval of the Institutional Animal Care and Use Committee at the University of Notre Dame.

### Adipokine array

The Proteome Profiler Mouse Adipokine Array Kit array kit #ARY013, R&D Systems, Minneapolis, MN) was used to profile the expression of 38 adipokines. Biologic triplicates of ascites (*n* = 3 per cohort) were centrifuged at 2000 RPM/min for 4 min at 4 °C to remove cells and debris. The supernatant was collected, aliquoted and frozen at -80 °C prior to use. The protein concentration of each of the three individual ascites samples was determined and 1 mg total protein was used to evaluate duplicate samples following the manufacturer’s specifications. Array results were detected with an Image Quant LAS 4000 and quantified using ImageQuant™ TL software. Data are reported using the arbitrary unit of “volume” that incorporates the signal area and signal intensity.

### MultiOmyx immunophenotyping of human ovarian tumors

Tumor blocks from women with high grade serous ovarian cancer with defined body mass index (BMI) were obtained from the Indiana University Simon Cancer Center tissue bank (Indianapolis, IN) with Institutional Review Board approval. Prior to multiplexing, sequential FFPE tumor tissue sections were stained with H&E according to standard protocols, reviewed by a pathologist, and 12 to 28 regions of interest were designated in each tumor for further analysis. Virtual pseudo-colored H&Es were generated from a combination of DAPI and Cy2 autofluorescence. Multiplexed immunofluorescence staining was performed using the MultiOmyx platform according to Gerdes et al. [25]. This technology was performed using a single 4 uM FFPE slide where for each staining round two cyanine dye-labeled (Cy3, Cy5) antibodies were paired together in a custom multiplex panel. For all rounds, staining signals were imaged and then followed by a dye inactivation step, enabling repeated rounds of staining. The proprietary deep learning-based workflow NeoLYTX was subsequently applied to identify individual cells and perform cell classification for each marker and the phenotype of each cell was then determined through co-expression analysis. Antibodies by staining order for the human panel were mouse anti-PanCK (PCK26, Sigma-Aldrich/AE1, BioScience), mouse anti-CD163 (EDHu-1, Bio-Rad), mouse anti-CD3 (F7.2.38, Dako), rabbit anti-PD-L1 (SP142, Abcam), rabbit anti-CD4 (EPR6855, Abcam), rabbit anti-PD-1 (EPR4877(2), Abcam), mouse anti-CD8 (C8/144B, Dako), mouse anti-FoxP3 (206D, BioLegend), rabbit anti-E Cadherin (EP700Y, Abcam), rabbit anti-vimentin (D21H3, Cell Signaling), mouse anti-HLA DQ/DR/DP (WR18, Novus), mouse anti-CD68 (KP1, BioLegend).

### Histologic analysis of murine tumors

Abdominal organs were fixed in 10% formalin and paraffin embedded for histological analysis. After deparaffinization, immuno-histochemical analysis for macrophage markers iNOS (rabbit, 1:1000 dilution, Abcam) and CD206 (rabbit, 1:6400 dilution, Abcam) was performed in serial sections as previously described [20]. Briefly, endogenous peroxidase activity was quenched with 3% hydrogen peroxide in methanol for 30 min prior to antigen retrieval by heat induction (99 °C in 10 mM Tris, 1 mM EDTA, pH 9.0, 20 min). Non-specific binding was blocked with 3% normal horse serum in PBS for 30 min. Sections were then incubated for 1 h at room temperature with primary antibody (at dilutions as indicated above) in 1% BSA in PBS. Peroxidase-conjugated secondary antibodies and peroxidase detection reagents were obtained from Vector Labs (Newark, CA). Slides were scanned into the eSlide Manager Database with the Aperio ScanScope CS (Leica Biosystems Inc., Buffalo Grove, IL, USA) and analyzed using the Aperio ePathology ImageScope pre-installed percent positive macro algorithm to quantify the number of DAB chromogen positive (brown) cells. Two slides were scanned per tumor and a minimum of 10 regions of interest per slide were evaluated. Statistical analysis was completed using Student’s *t*-test (Sigmaplot). The trichrome staining kit was purchased from Abcam (Waltham, MA) and used according to manufacturer’s specifications. Briefly, slides were deparaffinized and placed in Bouin’s Fluid, washed with water and incubated with Weigert’s Iron Hematoxylin. After washing, slides were incubated in Biebrich Scarlet/Acid Fuchsin solution, washed, and incubated in phosphomolybdic/phosphotungstic acid solution followed by Analine Blue solution. Rinsed slides were incubated in 1% acetic acid then dehydrated in alcohol prior to mounting. Slides were scanned using an Aperio Scanscope and processed with a modified Color Deconvolution Algorithm (v9) in Aperio ImageScope. Collagen appears as blue stain.

### Data availability

The data generated in this study are available within the article and its Supplementary data files.

## Results

### Host diet and chemotherapy response

**Fig. 1:**
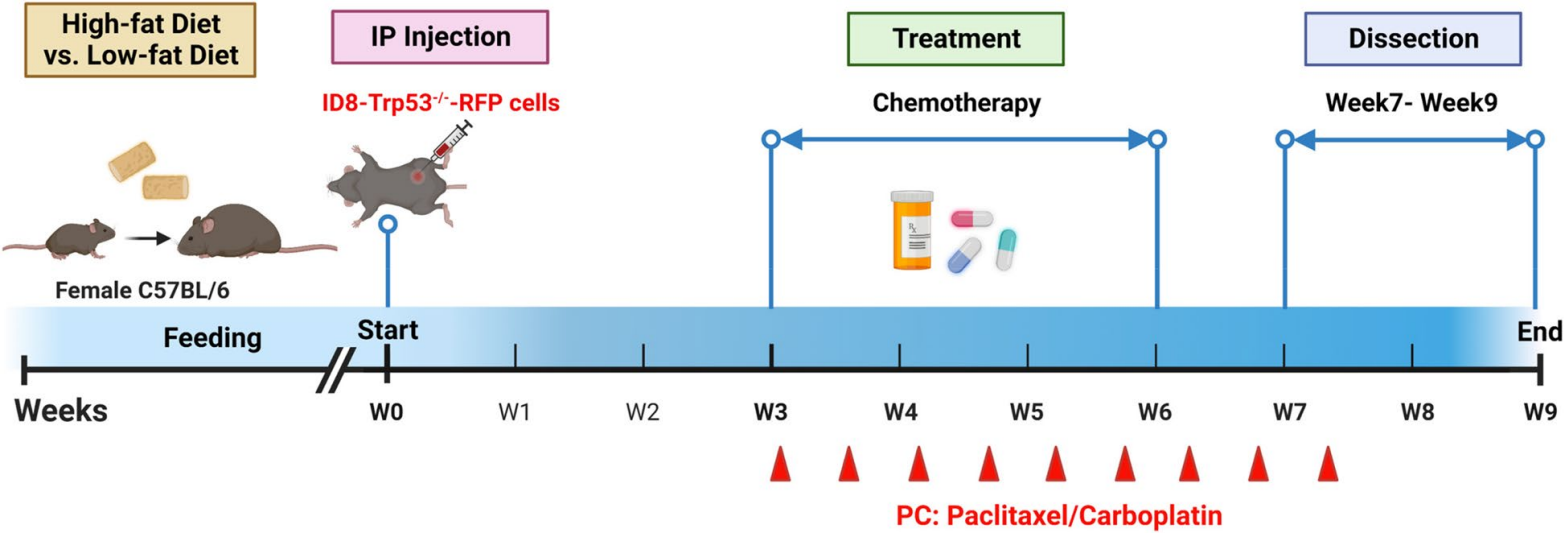
Experimental overview. Female mice were fed a control low fat diet (LFD) or a high fat diet (HFD) until a ~ 10 g difference in weight was attained prior to injection with murine ovarian cancer cells (ID8-Trp53^−/−^, W0). After three weeks (W3), mice were injected with weight-adjusted standard of care chemotherapy (paclitaxel and cisplatin; PC) twice weekly for a total of 6 or 9 cycles as indicated (red arrowheads). Dissection and quantitation of tumor burden occurred one week following cessation of chemotherapy (W7 or W9). Figure created with Biorender.com

To examine the potential impact of host weight on response to chemotherapy in a murine syngeneic model of ovarian cancer, we conducted a diet-induced obesity study in which mice were fed a control LFD (10% fat) or a HFD (45% fat) until a statistically significant difference in weight was achieved, then injected i.p. with RFP-tagged ID8-Trp53^−/−^ cells [22] (Fig. 1). In our initial cohort, after 16 weeks on a LFD or HFD, an average 7.5 g weight difference was observed prior to injection of tumor cells into *n* = 3 mice per cohort (Additional file 1: Figure 1A). Tumor-bearing mice were monitored by longitudinal in vivo imaging for development of equivalent tumor burden, then treated with 6 cycles of weight-adjusted chemotherapy [paclitaxel (6 mg/kg) and carboplatin (15 mg/kg)]. One week following the final chemotherapy treatment, mice were euthanized for end-point dissection. Using a midline incision to expose the peritoneal cavity, abdominal organs were imaged in situ prior to removal of organs for ex vivo imaging (Additional file 1: Figure 1B-D). Substantial remaining tumor burden was observed in images from HFD mice relative to mice on the LFD. Quantitation of overall tumor burden showed a 9-fold greater remaining tumor signal in intact HFD mice relative to the LFD cohort. Quantitation of organ-specific signal showed greater remaining tumor burden in the ovaries/uterus (10-fold), omentum (7.5-fold) and abdominal fat pad (9-fold). In this initial cohort, 2 out of 3 LFD mice had no detectable tumor burden on the ovaries/uterus and omentum while 1/3 LFD mice had no detectable tumor burden on the abdominal fat pad. In contrast, in the HFD cohort 3/3 mice had detectable remaining tumor burden on the omentum, ovaries/uterus, mesentery, and fat.

**Fig. 2:**
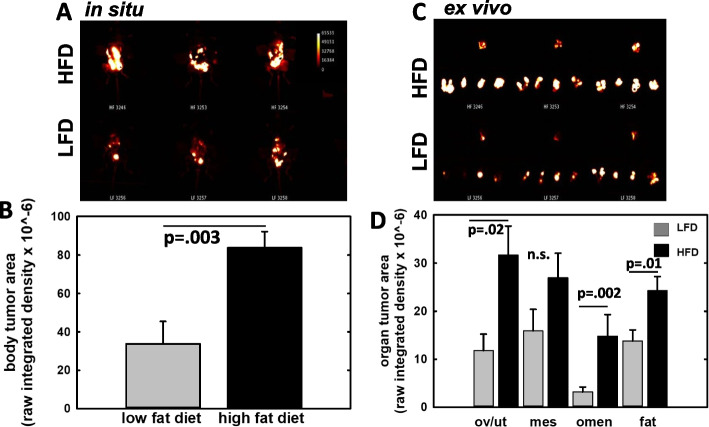
Analysis of residual tumor burden following chemotherapy. Mice on either control LFD (*n* = 11) or HFD (*n* = 10) were injected with 1 × 10^7^ RFP-tagged ID8-Trp53^−/−^ cells. After three weeks, mice were treated with 9 cycles of weight-adjusted paclitaxel (6 mg/kg) and carboplatin (15 mg/kg). One week following the last chemotherapy treatment, mice were sacrificed, the peritoneal cavity exposed with a midline incision, and RFP signal was imaged in situ using an Ivis Lumina to determine (**A**, **B**) total abdominal tumor burden. **C**, **D** Abdominal organs were then removed and imaged ex vivo to evaluate organ-specific tumor burden. Graphs show mean and standard error of the mean. Pairwise statistical analyses were conducted using Student’s t-test (Sigmaplot). n.s. = not significant (*p* > 0.05)

To evaluate whether increasing the number of chemotherapy cycles would improve response, a second cohort was examined. After 11 weeks on the HFD, an average 10.4 g weight difference was achieved prior to tumor cell injection (Additional file 1: Figure 2A) into *n* = 11 LFD and *n* = 10 HFD mice. Treatment duration was increased to 9 cycles of weight-adjusted paclitaxel and carboplatin. Analysis of remaining tumor burden one week following the end of treatment again showed substantial remaining tumor burden in the HFD cohort (Additional file 1: Fig. 2B-C; Fig. 2A-D). Quantitation of overall tumor burden showed a statistically significant 2.5-fold greater remaining tumor signal (*p* = 0.003) in intact HFD mice relative to the LFD cohort (Fig. 2A,B). Quantitation of organ-specific signal showed statistically significant differences with greater remaining tumor burden in the HFD ovaries/uterus (2.7-fold, *p* = 0.02), omentum (4.6-fold, *p* = 0.002) and abdominal fat pad (1.8-fold, *p* = 0.01) relative to LFD mice (Fig. 2C,D). Notably, 1 out of 11 LFD mice had no detectable tumor burden on the ovaries/uterus, mesentery, and fat while 3/11 LFD mice had no detectable tumor burden on the omentum. In contrast, in the HFD cohort 12/12 mice had detectable remaining tumor burden on the omentum, ovaries/uterus, mesentery, and fat.

### Immune profiling of human tumors from patients with high body mass index (BMI)

**Fig. 3:**
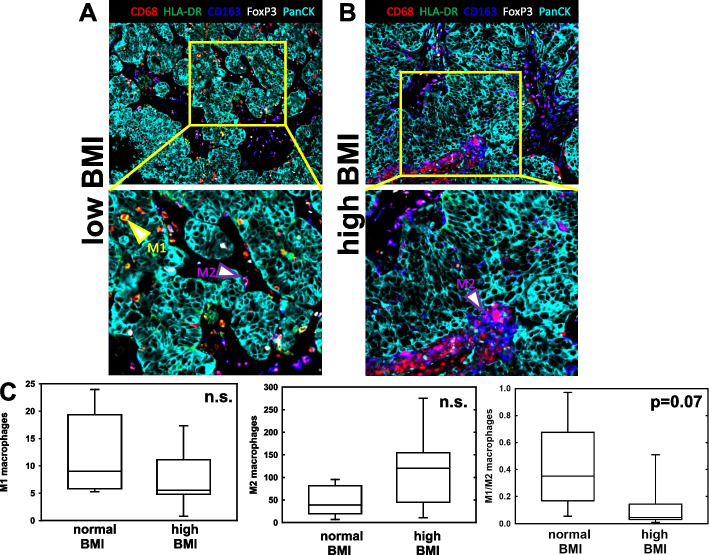
Evaluation of tumor-associated macrophage (TAM) staining in human ovarian tumors from patients with normal *vs* high body mass index (BMI). Tissues were stained using MultiOmyx technology as described. Regions of interest (12–28) were identified by a pathologist. M1 and M2 TAMS were quantified by applying the proprietary deep-learning based cell classification platform NeoLYTX to multiplexed images. **A**, **B** Representative color overlay images of tumors from patients with normal body mass index (BMI) and high BMI. Arrows indicate examples of M1 TAMs (yellow) and M2 TAMs (magenta). **C** Quantification of TAM staining data. Pairwise statistical analyses were conducted using Student’s t-test (Sigmaplot). n.s. = not significant (*p* > 0.05). Box and whisker plots show lower quartile, median, and upper quartile (box) and minimum/maximum values (whiskers)

To gain insight into host factors that may impact response to chemotherapy, a panel of human tumors from patients with defined body mass index (BMI) was evaluated. As obesity is well known to contribute to the development of low-grade inflammation of the visceral adipose tissue [26–28], we utilized MultiOmyx analysis to profile the immune landscape of these tumors. FFPE tissues (*n* = 13) were obtained from women with varying BMI (20.3–38.8 kg/m^2^, Table 1) and subjected to multiplexed immunofluorescence analysis with a panel of immuno-oncology biomarkers for immunophenotyping of human immune cells (Table 2). Images were analyzed by applying the deep-learning based cell classification platform NeoLYTX. Of the 13 tissues analyzed, 6 were from patients with normal BMI < 30 (range 20.3–22.4) and 7 from patients with high BMI > 30 (range 32.8–38.8). While significant differences were not detected for the majority of immune phenotypes evaluated (Additional file 1: Figs. 3 and 4), we observed a decrease in M1 macrophages and an increase in M2 macrophages in high BMI patients, resulting in a 3.2-fold decrease in the M1/M2 ratio with moderate significance (*p* = 0.07) (Fig. 3).

**Table 1:** Summary of patient data. Tumor tissues were obtained from patients with normal body mass index (BMI, patients 1–6) or high BMI (patients 7–13). The average BMI of the ‘normal BMI’ group was 21.27 ± 0.32 while the average BMI of the ‘high BMI’ group was 35.60 ± 0.90 (*p* < 0.001). The average age of the ‘normal BMI’ group was 66.67 ± 3.65 while the average age of the ‘high BMI’ group was 61.74 ± 3.53 (*p* = 0.35)

Patient Information				
**Patient #**	**BMI (kg/m**^**2**^**)**	**Age**	**Diagnosis**	**Stage**
1	20.3	79	Adenocarcinoma, NOS	ND
2	20.5	56	Papillary serous adenocarcinoma	IIIC
3	21.3	65	Papillary serous carcinoma	ND
4	21.4	62	Serous carcinoma	IIIC
5	21.7	76	Serous adenocarcinoma	IIIC
6	22.4	62	Papillary serous adenocarcinoma	ND
7	32.8	55	Serous carcinoma	IIIC
8	33.7	70	Serous adenocarcinoma	IIIC
9	33.7	78	Papillary serous carcinoma	ND
10	35.5	53	Serous carcinoma	ND
11	36.3	63	Serous carcinoma	IIIC
12	38.4	59	Papillary serous carcinoma	ND
13	38.8	54	Papillary serous carcinoma	ND

*ND* No data, *NOS* Not otherwise specified

**Table 2:** Phenotyping of human immune cells

CO-EXPRESSION	PHENOTYPE
CD3 + CD4 +	T helper
CD3 + CD4 + FoxP3 +	T regulator
CD3 + CD8 +	T cytotoxic
CD3 + CD4 + PD1 +	T helper PD1 +
CD3 + CD8 + PD1 +	T cytotoxic PD1 +
CD68 + HLA-DR +	M1 TAM
CD68 + CD163 +	M2 TAM
CD68 + PDL1 +	PDL1 + TAM
PanCK + PDL1 +	Tumor + PDL1

### Analysis of murine tumor tissues from LFD and HFD mice

**Fig. 4:**
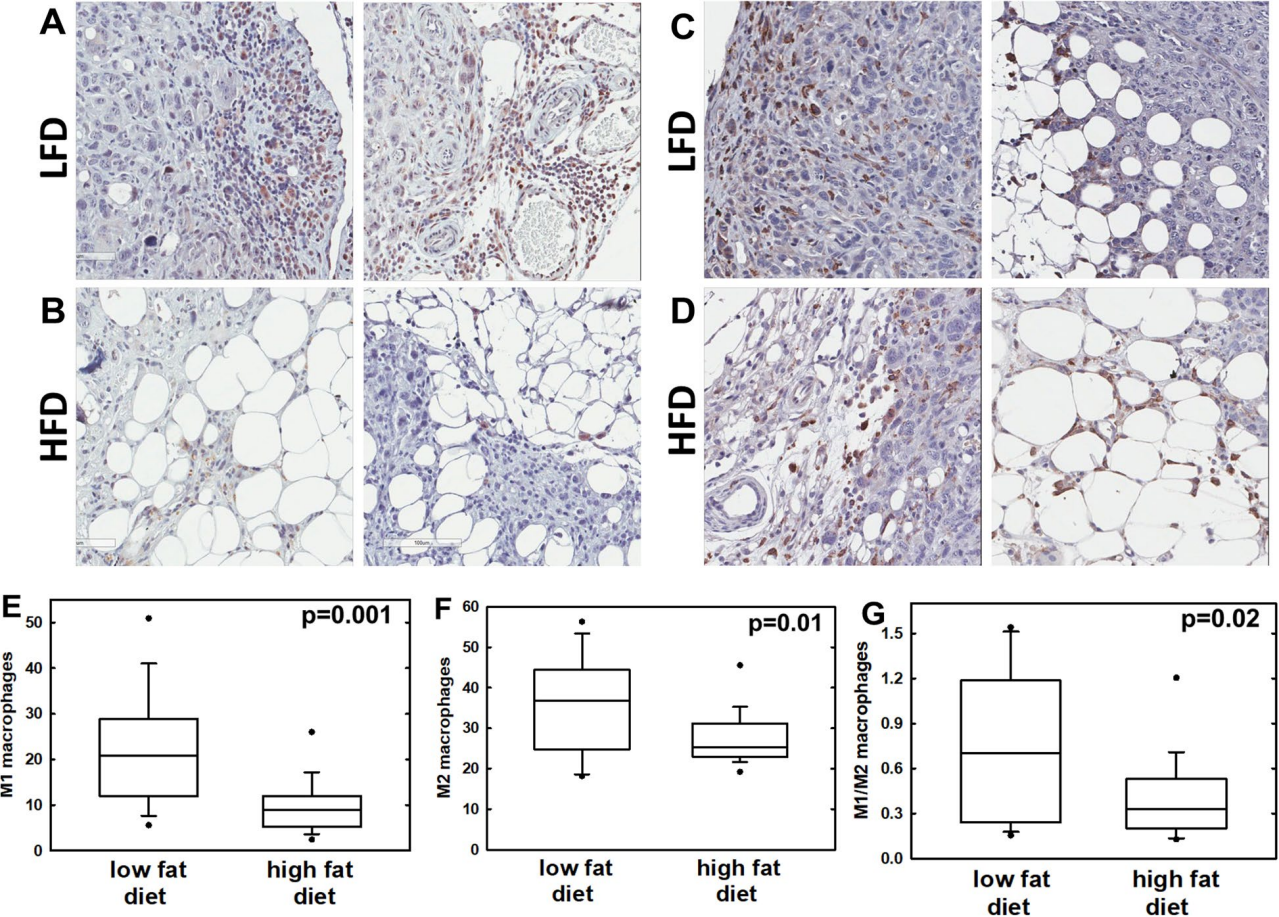
Evaluation of TAM staining in murine tumors from low fat diet (LFD) and high fat diet (HFD) mice. **A**, **B** Sections were stained for M1 TAMs using anti-iNOS antibodies (1:1000 dilution) followed by peroxidase-conjugated secondary antibody and peroxidase detection. **C**, **D** Sections were stained for M2 TAMS using anti-CD206 antibodies (1:6400 dilution) followed by peroxidase-conjugated secondary antibody and peroxidase detection. **E**–**G** Stained sections were quantified using an Aperio Scanscope and analyzed using ePathology ImageScope software percent positive macro algorithm to quantify the number of DAB chromogen positive (brown) cells. Two slides were scanned per tumor and a minimum of 10 regions of interest per slide were evaluated. Statistical analysis was completed using Student’s *t*-test (Sigmaplot). Box and whisker plots show lower quartile, median, and upper quartile (box) and minimum/maximum values (whiskers)

Based on the findings above with human tumor tissues, murine tissues from the LFD and HFD cohorts were subjected to immunohistochemical analysis to evaluate M1 and M2 macrophage profiles. Similar to results obtained with human tumors from high BMI patients, mice on the HFD protocol demonstrated a significantly decreased M1/M2 ratio (2-fold decrease, *p* = 0.02) (Fig. 4). No significant differences were observed in the remaining immune phenotypes evaluated in murine tissues (Additional file 1: Table 1; Additional file 1: Fig. 5).

To examine differentially expressed adipokines that may potentially contribute to altered macrophage polarization, an adipokine profiler array was performed on ascites from *n* = 3 LFD and *n* = 3 HFD mice. Only FGF21 was significantly increased in the ascites from HFD mice (12.5-fold increase relative to ascites from LFD mice, *p* = 0.001) (Table 3). Adipokines that were expressed at significantly higher levels in the ascites from LFD mice include adiponectin and IGFBP-2, -3, -5, and -6 (Table 3). Additional adipokine profiles are shown in Additional file 1: Table 2.

**Table 3:** Adipokine array analysis of ascites from mice on low fat diet (LFD) or high fat diet (HFD). A Proteome Profiler Mouse Adipokine Array was used to profile the expression of adipokines in ascites (*n* = 3 individuals per cohort, duplicate samples). Samples were normalized for protein content and analyzed according to manufacturer’s specifications. Array results were detected with an Image Quant LAS 4000 and quantified using ImageQuant™ TL software. Data are reported using the arbitrary unit of “volume” that incorporates the signal area and signal intensity

**Adipokine**	**Low fat diet ascites** (volume units × 10^–5^)	**High fat diet ascites** (volume units × 10^–5^)	***P* value**
Adiponectin	4.98 +/- 0.29	3.35 +/- 0.14	0.001
FGF21	0.06 +/- 0.01	0.75 +/- 0.14	0.002
IGFBP-2	6.32 +/- 0.94	4.51 +/- 0.85	0.006
IGFBP-3	3.36 +/- 0.08	2.46 +/- 0.22	0.004
IGFBP-5	0.24 +/- 0.08	0.06 +/- 0.01	0.037
IGFBP-6	3.44 +/- 0.05	2.41 +/- 0.83	0.002

*Abbreviations*: *FGF21* Fibroblast growth factor 21, *IGFBP* Insulin-like growth factor binding protein

**Fig. 5:**
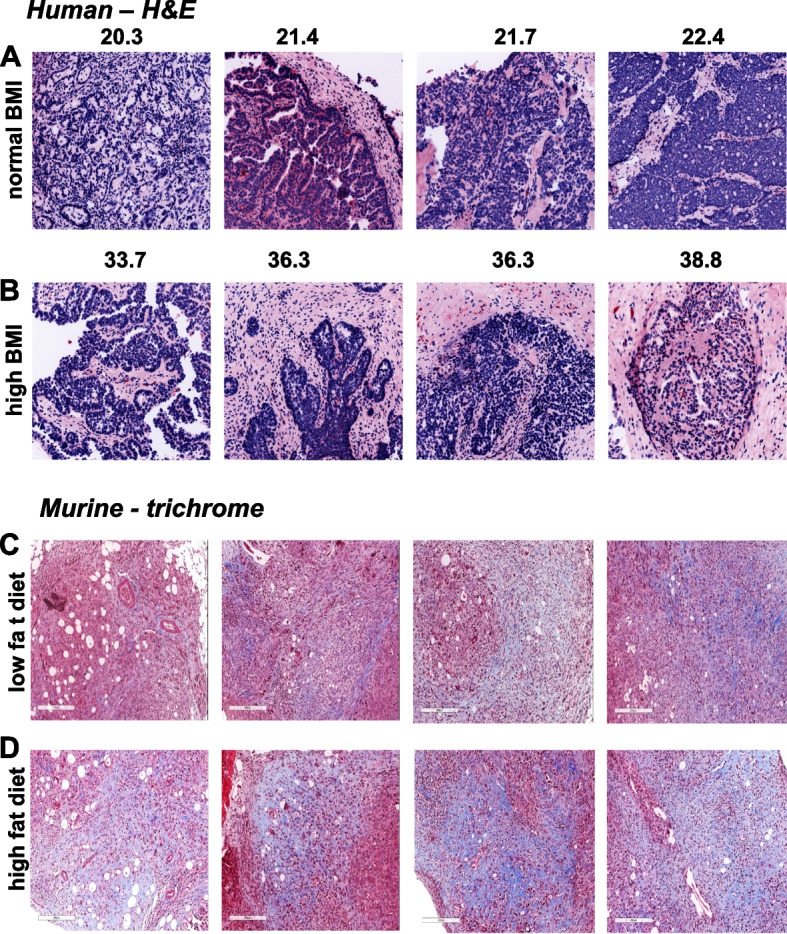
Tumor-associated fibrosis. **A**, **B** Hematoxylin and eosin (H&E) staining of human ovarian tumor tissues. Representative tissues from patients with (**A**) normal BMI (*n* = 6) or (**B**) high BMI (*n* = 7) were stained using H&E. Fibrotic tissue (pink) is more evident in the high BMI cohort. **C**, **D** Trichrome staining of tumors from LFD and HFD mice. Tumor sections from mice on (**C**) LFD (*n* = 11) or (**D**) HFD (*n* = 10) were stained using trichrome reagents according to the manufacturer’s specifications. Representative images are shown. Collagen staining (blue) is more evident in the HFD cohort. Scale bar 200 μm

Evaluation of human tumors showed enhanced tumor-associated fibrosis in the specimens from women with BMI > 30 (Fig. 5A,B). Tumor sections from mice on LFD vs HFD were stained using Masson’s trichrome to visualize collagen deposition. Relative to tumors grown in LFD mice, enhanced areas of fibrosis were found in tumors from HFD mice (Fig. 5C,D).

## Discussion

The World Health Organization has identified obesity as a non-infectious and non-communicable pandemic that significantly impacts disease-related morbidity and death [6]. The most recent National Health and Nutrition Examination Survey data show the prevalence of obesity (BMI = 30 kg/m^2^ or above) in U.S. women over age 20 as 41.8%, with 11.7% exhibiting severe obesity (BMI = 40 kg/m^2^ and above) [29]. Evaluation of data from 10 countries with an obesity prevalence over 25% show that mortality rates for women with ovarian cancer have not decreased, but rather have remained stable or are increased [6]. Another global study showed increasing incidence in younger women (aged 15–40) in some countries that was correlated with high rates of obesity and metabolic syndrome [1]. Obesity and overweight can result in post-operative complications which can delay adjuvant therapies, thereby affecting overall prognosis [30]. In addition, obesity may also impact metastatic success and response to therapy. This is supported by a study characterizing a “poor outcome” cohort of HGSOC patients whose tumors express an upregulated cluster of obesity- and lipid metabolism-related genes. These women have significantly reduced progression-free and overall survival relative to women with the same mutational profile but lacking this gene cluster [17]. Moreover, in a study of women undergoing secondary cytoreductive surgery for recurrent ovarian cancer, BMI was an independent predictor of poor survival, further suggesting an effect of host weight on tumor biology and/or treatment response [31]. These epidemiologic data are supported by the findings of the current pre-clinical study, showing a poor response to standard-of-care chemotherapy in mice on a high fat diet. Our data show substantial remaining tumor burden in HFD mice following 6–9 cycles of weight-adjusted standard of care paclitaxel and carboplatin chemotherapy. These tumors also exhibited a significant decrease in the M1/M2 macrophage ratio and enhanced tumor fibrosis. A similar decrease in M1/M2 macrophage staining and enhanced fibrosis was also observed in tumors from HGSOC patients with high BMI. While a direct link between tumor-associated macrophages and fibrosis was not evaluated in this study, it is interesting to note that recent studies have demonstrated that macrophages, in addition to cancer-associated fibroblasts, contribute to collagen deposition and tumor fibrosis in ovarian and colorectal cancers [32, 33]. Specifically, TAMs have been shown to directly participate in collagen deposition, cross-linking, and linearization [33].

Obesity has been shown to alter extracellular matrix deposition, and this desmoplasia can hamper the response to chemotherapy [34]. In the current study, enhanced tumor-associated fibrosis was observed both in human tumor tissues from high BMI patients and in tumors from mice fed a HFD. Therapeutic targeting of tumor-associated fibrosis was recently shown to enhance the response to chemotherapy in a murine model of ovarian cancer [35]. In this study, mice treated with the anti-hypertensive agent Losartan in combination with pegylated liposomal doxorubicin demonstrated reduced intratumoral collagen and hyaluronan content relative to single agent controls. Moreover, a retrospective analysis of ovarian cancer patients taking similar agents in addition to standard of care chemotherapy showed a significant increase in overall survival (63 mo relative to 33 mo in controls) [35]. Another pre-clinical report showed that antibody targeting of the protein MFAP5 reduced fibrosis in ovarian tumors in mice and enhanced response to the chemotherapeutic agent paclitaxel. These investigators also identified a “fibrotic gene signature” in human tumors predictive of significantly reduced survival relative to patients whose tumors had low expression of this gene signature (19 *vs* 33 mo, respectively) [36]. A third study investigated age-associated ovarian fibrosis both in murine models and in human ovaries, demonstrating enhanced deposition of anisotropic collagen in aged ovaries [37]. In the human cohort the use of metformin, which has been shown to reduce or prevent fibrosis in pre-clinical models, was strongly associated with reduced fibrosis. Interestingly, fibrotic ovaries showed increased M2-macrophages relative to non-fibrotic ovaries or ovaries of metformin users, indicating a correlation between ovarian fibrosis and M2-polarization of macrophages. While the above studies did not evaluate the impact of obesity in pre-clinical murine models or retrospective analyses of patient data, these collective results suggest that an anti-fibrosis therapeutic strategy may enhance chemotherapeutic efficacy in patients with high BMI or in pre-clinical models of diet-induced obesity.

To evaluate potential factors that may influence macrophage polarization, adipokine expression was evaluated. FGF21 was the only adipokine, expression of which was elevated in the ascites of mice on a HFD. A predominant function of FGF21 is to stimulate insulin-independent glucose uptake. Little is known about the role of FGF21 in ovarian cancer; however a recent report identified FGF21 as a highly significantly upregulated gene when comparing cisplatin-resistant human ovarian cancer cells (A2780CP) to the parental cell line (A2780) [38]. Modulation of FGF21 levels by overexpression or siRNA silencing confirmed that FGF21 overexpression induces chemoresistance in ovarian cancer cells [38]. These data suggest that FGF21-mediated chemoresistance may contribute mechanistically to the observed tumor burden remaining in HFD mice after chemotherapy treatment. Additional murine studies showed that FGF21 expression alters macrophage polarization in liver and adipose tissue, resulting in a decreased M1/M2 ratio [39, 40]. Together these data suggest that additional studies on expression of FGF21 in ovarian cancer and its role in therapeutic response are warranted.

Adipokine array data also showed a significant decrease in adiponectin in ascites from HFD mice relative to LFD mice. A role for adiponectin in macrophage polarization has been reported, although effects appear to differ based on the specific cancer type and animal model under investigation [41]. However previous studies in mice and humans have shown that decreased serum adiponectin levels are associated with obesity, leading to hyperinsulinemia and insulin resistance [41, 42]. As a consequence, levels of IGFPB-1 and -2 are decreased, thereby increasing IGF1 bioavailability. Together insulin and IGF1 upregulate VEGF and vascularity [41]. These observations are of interest in light of the current study showing decreased levels of multiple IGFBPs (IGFBP-2, -3, -5, and -6) in HFD mice. This is consistent with an observation from our previous study showing enhanced tumor vascularity in mice fed on a western diet (40% fat) relative to control diet mice [20]. A more detailed mechanistic analysis of the adiponectin/IGFBP/VEGF axis in regulation of vessel density in tumors of HFD mice is underway.

Immunotherapy incorporating immune checkpoint inhibitors (ICIs) has proven highly effective in treating many cancers [43]. However, despite relatively high levels of tumor infiltrating lymphocytes, a number of clinical trials have shown a therapeutic efficacy of only 10–15% in trials using ICIs in ovarian cancer patients, suggesting that other components of the tumor microenvironment contribute to this treatment failure [43, 44]. While obesity is typically associated with dysregulated immune responses, termed “inflammaging” [45], an interesting recent study [46] reported that obesity enhanced efficacy of ICI blockade in both pre-clinical murine models of obesity and melanoma and in high BMI human melanoma patients. In this study, obesity was found to induce T cell aging, characterized by higher PD-1 expression which rendered tumors more responsive to checkpoint blockade therapies [43]. Data in the current study show no changes in PD-1 expression levels on either helper T cells or cytotoxic T cells when comparing ovarian cancer patients of high BMI to those of low BMI (Supplemental data), suggesting that targeting other aspects of the tumor immune microenvironment may be preferential.

Tumor-associated macrophages (TAMs) are the most abundant immune cell type in the ovarian tumor microenvironment and can be broadly characterized as classically activated pro-inflammatory (M1) or alternatively activated anti-inflammatory (M2) based on surface marker expression and cytokine and chemokine profiles [44, 47]. It has previously been reported that, while TAM density increases with increasing stage and grade, an overall decrease in M1/M2 ratio was observed with increasing cancer stage [48]. This finding was confirmed by additional studies including a meta-analysis of 794 patients, showing that a high M1/M2 ratio was associated with more favorable overall survival and was predictive of better progression-free survival [49]. Moreover, a high M1/M2 ratio also associated with an improvement in platinum-free interval [50]. Given these data and the current findings, of interest are potential therapeutic strategies that target TAM survival or induce repolarization of M2 to M1 TAMs. One such candidate is trabectedin, the lead compound of ecteinascidins, that was approved by the U.S. Food and Drug Administration in 2015 (Yondelis) for treatment of unresectable or metastatic liposarcoma or leiomyosarcoma and by the European Union for treatment of relapsed platinum-sensitive ovarian cancer in 2009 [49]. This drug has a unique dual mechanism of action wherein it induces cytotoxicity by forming adducts with minor groove DNA, inducing single-strand and double-strand breaks, cell cycle arrest and apoptosis [51–53]. Additionally, trabectedin modulates the tumor immune microenvironment by selectively inducing apoptosis of monocytes/macrophages via caspase-8 activation, with an associated reduction in inflammatory cytokines/chemokines and angiogenesis [52]. Interestingly, trabectedin retained anti-tumor efficacy in a pre-clinical model in which trabectedin-resistant IGROV cells were developed in vitro and xenografted in vivo [52]. Even though these tumor cells were unresponsive to trabectedin in vitro*,* neoplastic growth in vivo was significantly reduced, providing further evidence that targeting TAMS in the tumor microenvironment represents an important component of the anti-tumor activity of this drug. Of interest would be a retrospective evaluation of data from completed clinical trials of ovarian cancer patients treated with trabectedin to assess whether BMI is statistically associated with therapeutic efficacy, as well as the targeted recruitment of high BMI patients to future trials with this agent.

## Conclusion

The prevalence of obesity is increasing worldwide. Obesity has an adverse effect on survival of women with ovarian cancer. The current pre-clinical data highlight potential mechanisms by which tumors in obese or HFD hosts may be less chemoresponsive relative to lean or LFD hosts via altered tumor-associated macrophage polarization and enhanced fibrosis. These data suggest that combination therapies targeting macrophage polarization and/or fibrosis may enhance efficacy of standard-of-care chemotherapy in the obese host and improve survival of women with ovarian cancer.

## Supplementary Information

**Additional file 1: **Additional methods. **Additional Table 1.** Phenotyping of murine immune cells. **Additional Table 2.** Additional adipokine array analysis of ascites from mice on low fat diet (LFD) or high fat diet (HFD). **Additional Fig. 1.** Pilot pre-clinical trial cohort of high fat diet (HFD) and chemotherapy response. **Additional Fig. 2.** Complete data from pre-clinical trial of HFD and chemotherapy response. **Additional Fig. 3.** Evaluation of immune cell staining in human ovarian tumors from patients with normal *vs* high body mass index (BMI). **Additional Fig. 4.** Additional immune cell and epithelial-mesenchymal transition (EMT) marker staining in human ovarian tumors from patients with normal *vs* high BMI. **Additional Fig. 5.** Evaluation of immune cell staining in murine tumors from mice on a control low fat diet (LFD) or high fat diet (HFD).

## Data Availability

The data generated in this study are available within the article and its supplementary data files.

## Abbreviations

BMI: Body mass index
DIO: Diet-induced obesity
FGF21: Fibroblast growth factor 21
HFD: High fat diet
HGSOC: High grade serous ovarian cancer
IGF1: Insulin-like growth factor 1
IGFBP-1: Insulin-like growth factor binding protein 1
i.p.: Intra-peritoneal
LFD: Low fat diet
OvCa: Ovarian cancer
RFP: Red fluorescent protein
ROI: Region of interest
SOC: Standard of care
TAM: Tumor-associated macrophage
