# Supplementary material for: Host obesity alters the ovarian tumor immune microenvironment and impacts response to standard of care chemotherapy
Source: J Exp Clin Cancer Res. 2023 Jul 12;42:165. doi: 10.1186/s13046-023-02740-y (PMC10337170; doi:10.1186/s13046-023-02740-y)

Liu et al., ADDITIONAL TEXT

ADDITIONAL METHODS

MultiOmyx immunophenotyping of murine tumors. Multiplexed immunofluorescence staining was performed using the MultiOmyx platform according to Gerdes et al [25] on tumor tissues from control low fat diet (LFD) or high fat diet (HFD) mice. This technology was performed using a single 4 uM FFPE slide where for each staining round two cyanine dye-labeled (Cy3, Cy5) antibodies were paired together. A custom multiplex 9-marker panel was created, except for CD68 and PD-L1 in the murine panel where they were applied as primary-secondary antibodies followed by incubation with a species-specific secondary antibody conjugated to cyanine 5 or 3 (Cy5 or Cy3). For all rounds, staining signals were imaged and then followed by a dye inactivation step, enabling repeated rounds of staining. The proprietary deep learning-based workflow NeoLYTX was subsequently applied to identify individual cells and perform cell classification for each marker and the phenotype of each cell was then determined through co-expression analysis. Antibodies by staining order for the murine panel were rabbit anti-CD68 (polyclonal, Abcam), rat anti-PD-L1 (MIH6, BioLegend), rabbit anti-CD3 (D4V8L, BioLegend), rabbit anti-FoxP3 (D6O8R, BioLegend), mouse anti-PanCK (PCK26, Sigma-Aldrich/AE1, BioScience), rabbit anti-CD44 (EPR18668, Abcam), rabbit anti-CD4 (D7D2Z, Cell Signaling), rabbit anti-CD8 (D4W2Z, Cell Signaling), rabbit anti-vimentin (D21H3, Cell Signaling).

ADDITIONAL TABLES

Additional Table 1. Phenotyping of Murine Immune Cells	
**CO-EXPRESSION**	**PHENOTYPE**
CD3+CD4+	T helper
CD3+CD4+foxP3+	T regulator
CD3+CD8+	T cytotoxic
CD68+CD44+	TAM CD44+
CD68+PDL1+	TAM PDL1+
CD68+VIM+	TAM VIM
PanCK+PDL1+	Tumor PDL1+
PanCK+CD44+	Tumor CD44+

Additional Table 2. Additional adipokine array analysis of ascites from mice on low fat diet (LFD) or high fat diet (HFD). A Proteome Profiler Mouse Adipokine Array was used to profile the expression of adipokines in ascites (n=3 per cohort, duplicate samples). Samples were normalized for protein content and analyzed according to manufacturer's specifications. Array results were detected with an Image Quant LAS 4000 and quantified using ImageQuant™ TL software. Data are reported using the arbitrary unit of "volume" that incorporates the signal area and signal intensity. Table shows raw data (duplicate analyses of three individual ascites samples) and mean/standard error of the mean for adipokines that were significantly differentially expressed in ascites from LFD *vs* HFD mice. Student's t-test (Sigmaplot) was used to calculate p values. **Abbreviations:** ANGPT-3, angiopoietin-3; APN, adiponectin; CRP, C-reactive protein; DPPIV, dipeptidyl peptidase IV; FGF21, fibroblast growth factor 21; IGFBP, insulin-like growth factor binding protein; ICAM-1, inter-cellular adhesion molecule 1; MCP1, monocyte chemo-attractive protein 1; M-CSF, macrophage colony stimulating factor; PENT-3, pentraxin 3; RBP4, retinol binding protein-4.

**Adipokine**	**Raw data low fat diet ascites** (volume unitsx10^{-5})	**Mean+/-S.E.M. low fat diet ascites** (volume units x 10^{-5})	**Raw data high fat diet ascites** (volume unitsx10^{-5})	**Mean+/- S.E.M. high fat diet ascites** (volume units x 10^{-5})	**P value**
ANGPT-L3	0.43<br>0.43<br>0.58<br>0.57<br>0.33<br>0.30	0.44+/-0.12	0.25<br>0.27<br>0.13<br>0.13<br>0.11<br>0.10	0.15+/-0.07	<0.001
APN	5.97<br>5.77<br>4.76<br>4.38<br>4.62<br>4.40	4.98+/-0.29	3.78<br>3.57<br>3.44<br>3.42<br>3.00<br>2.89	3.35+/-0.14	0.001
CRP	1.70<br>1.66<br>1.65<br>1.55<br>1.55<br>1.54	1.61+/-0.07	1.32<br>1.39<br>0.82<br>0.81<br>0.87<br>0.88	1.01+/-0.26	<0.001
DPPIV	0.41<br>0.42<br>0.40<br>0.41<br>0.29<br>0.31	0.37+/-0.06	0.37<br>0.37<br>0.15<br>0.14<br>0.09<br>0.10	0.21+/-0.13	0.02
ENDOCAN	0.19<br>0.18<br>0.24<br>0.21<br>0.22<br>0.22	0.21+/-0.02	0.03<br>0.04<br>0.06<br>0.08<br>0.06<br>0.06	0.06+/-0.02	<0.001
FETUIN A	0.27<br>0.24<br>0.17<br>0.17<br>0.06<br>0.08	0.16+/-0.08	0.10<br>0.10<br>0.05<br>0.04<br>0.15<br>0.27	0.06+/-0.04	0.01
FGF21	0.06	0.06+/-0.01	0.96	0.75+/-0.14	0.002

	0.09<br>0.03<br>0.03<br>0.06<br>0.08		0.98<br>0.96<br>0.96<br>0.33<br>0.33		
ICAM-1	5.20<br>5.24<br>4.70<br>4.80<br>3.72<br>3.84	4.58+/-0.66	3.89<br>3.86<br>3.73<br>3.69<br>3.30<br>3.03	3.59+/-0.34	0.008
IGFBP-2	6.25<br>6.16<br>6.24<br>5.88<br>5.29<br>5.08	6.32+/-0.94	5.59<br>5.47<br>4.44<br>4.24<br>3.73<br>3.61	4.51+/-0.85	0.006
IGFBP-3	3.30<br>3.49<br>3.57<br>3.52<br>3.23<br>2.95	3.36+/-0.08	3.16<br>3.17<br>2.11<br>2.22<br>2.01<br>2.11	2.46+/-0.22	0.004
IGFBP-5	0.46<br>0.47<br>0.17<br>0.16<br>0.08<br>0.07	0.24+/-0.08	0.05<br>0.05<br>0.05<br>0.03<br>0.08<br>0.08	0.06+/-0.01	0.037
IGFBP-6	3.31<br>3.38<br>3.53<br>3.61<br>3.41<br>3.39	3.44+/-0.05	3.15<br>3.15<br>2.15<br>2.14<br>1.91<br>1.95	2.41+/-0.83	0.002
MCP1	0.51<br>0.45<br>0.33<br>0.27<br>0.17<br>0.16	0.32+/-0.14	0.14<br>0.13<br>0.09<br>0.11<br>0.21<br>0.28	0.16+/-0.07	0.04
M-CSF	0.31<br>0.31<br>0.22<br>0.20<br>0.09	0.20+/-0.09	0.14<br>0.11<br>0.05<br>0.07<br>0.08	0.09+/-0.03	0.02

	0.10		0.08		
PENT-3	0.47<br>0.49<br>0.49<br>0.48<br>0.38<br>0.37	0.45+/-0.05	0.24<br>0.24<br>0.25<br>0.25<br>0.24<br>0.22	0.24+/-0.01	<0.001
RBP4	0.14<br>0.13<br>0.09<br>0.04<br>0.07<br>0.08	0.09+/-0.04	0.08<br>0.08<br>0.04<br>0.03<br>0.03<br>0.03	0.04+/-0.03	0.04
SERPIN E1	1.75<br>1.62<br>1.49<br>1.45<br>1.18<br>1.20	1.45+/-0.23	1.22<br>1.19<br>1.21<br>1.17<br>1.04<br>1.00	1.14+/-0.09	0.01

ADDITIONAL FIGURE LEGENDS

Additional Fig. 1. Pilot pre-clinical trial cohort of high fat diet (HFD) and chemotherapy response. (A) Mice (n=3/cohort) were fed on a low fat diet (LFD, grey symbols) or a HFD (black symbols) for 16 weeks until a 7.6g difference in weight was achieved, then injected with $1x10^7$ RFP-tagged ID8-$Trp53^{-/-}$ cells. After three weeks, mice were treated with 6 cycles of weight-adjusted paclitaxel (6mg/kg) and carboplatin (15 mg/kg). (B) Template showing position of organs in panel D. (C) One week following the last chemotherapy treatment, mice were sacrificed, the peritoneal cavity exposed with a midline incision, and RFP signal was imaged *in situ* using an Ivis Lumina. (D) Abdominal organs were then removed and imaged *ex vivo* to evaluate organ-specific tumor burden. Results in C,D show substantial remaining tumor burden following chemotherapy in mice on the HFD.

Additional Fig. 2. Complete data from pre-clinical trial of HFD and chemotherapy response. (A) Mice were fed on a (grey symbols, n=11) or a HFD (black symbols, n=10) for 11 weeks until an average 10.4g difference in weight was achieved, then injected with $1x10^7$x RFP-tagged ID8-$Trp53^{-/-}$ cells. After three weeks, mice were treated with 9 cycles of weight-adjusted paclitaxel (6mg/kg) and carboplatin (15 mg/kg). (B) Template showing position of organs in panel D. (C) One week following the last chemotherapy treatment, mice were sacrificed, the peritoneal cavity exposed with a midline incision, and RFP signal was imaged *in situ* using an Ivis Lumina. (D) Abdominal organs were then removed and imaged *ex vivo* to evaluate organ-specific tumor burden. Results in C,D show substantial remaining tumor burden following chemotherapy in mice on the HFD. Data are quantified in Fig. 2 in the main manuscript.

Additional Fig. 3. Evaluation of immune cell staining in human ovarian tumors from patients with normal *vs* high body mass index (BMI). Tissues were stained using MultiOmyx

technology as described. Positive staining was quantified by applying the proprietary deep-learning based cell classification platform NeoLYTX to multiplexed images. Representative color overlay images of tumors from patients with (A) normal body mass index (BMI) and (B) high BMI were stained for T helper cells (yellow arrowhead) and cytotoxic T lymphocytes (magenta arrowhead). (C) Quantification of T helper cells (normal BMI vs high BMI, p=0.85). (D) Quantification of cytotoxic T cells (normal BMI vs high BMI, p=0.95). (E,F) Representative color overlay images of tumors from patients with (E) normal BMI and (F) high BMI were stained for PD-1+ T helper cells (yellow arrowhead) and PD-1+cytotoxic T lymphocytes (magenta arrowhead). (G) Quantification of PD-1+ T helper cells (normal BMI vs high BMI, p=0.66). (H) Quantification of PD-1+ cytotoxic T cells. (normal BMI vs high BMI, p=0.76). n=6 normal BMI, n=7 high BMI.

Additional Fig. 4. Additional immune cell and epithelial-mesenchymal transition (EMT) marker staining in human ovarian tumors from patients with normal *vs* high BMI. Tissues were stained using MultiOmyx technology as described. Positive staining was quantified by applying the proprietary deep-learning based cell classification platform NeoLYTX to multiplexed images. Representative color overlay images of tumors from patients with (A) normal BMI and (B) high BMI were stained for T regulatory cells (magenta arrowhead). (C) Quantification of T regulatory cells (normal BMI vs high BMI, p=0.95). Representative color overlay images of tumors from patients with (D) normal BMI and (E) high BMI were stained for PD-1+ tumor associated macrophages (TAMs) (green arrowhead). (F) Quantification of PD-1+ TAMs (normal BMI vs high BMI, p=0.16). Representative color overlay images of tumors from patients with (G) normal BMI and (H) high BMI were stained for E-cadherin (green) and vimentin (red). (H) Quantification of E-cadherin/vimentin staining (normal BMI vs high BMI, p=0.24). n=6 normal BMI, n=7 high BMI.

Additional Fig. 5. **Evaluation of immune cell staining in murine tumors from mice on a control low fat diet (LFD) or high fat diet (HFD).** Tissues were stained using MultiOmyx technology as described. Positive staining was quantified by applying the proprietary deep-learning based cell classification platform NeoLYTX to multiplexed images. Representative color overlay images of tumors from mice on (A,C) LFD and (B,D) HFD were stained for T helper cells (yellow arrowhead), cytotoxic T lymphocytes (magenta arrowhead), T regulatory cells (white arrowhead) or PD-L1+ TAMs (green arrowheads). (E) Quantification of T helper cells (LFD *vs* HFD, p=0.66). (F) Quantification of T regulatory cells (LFD *vs* HFD, p=0.69) (G) Quantification of cytotoxic T cells (LFD *vs* HFD, p=0.69). (H) Quantification of PD-L1+ TAMs (LFD *vs* HFD, p=0.56). n=10/cohort.

Liu et al., Addl. Fig. 1

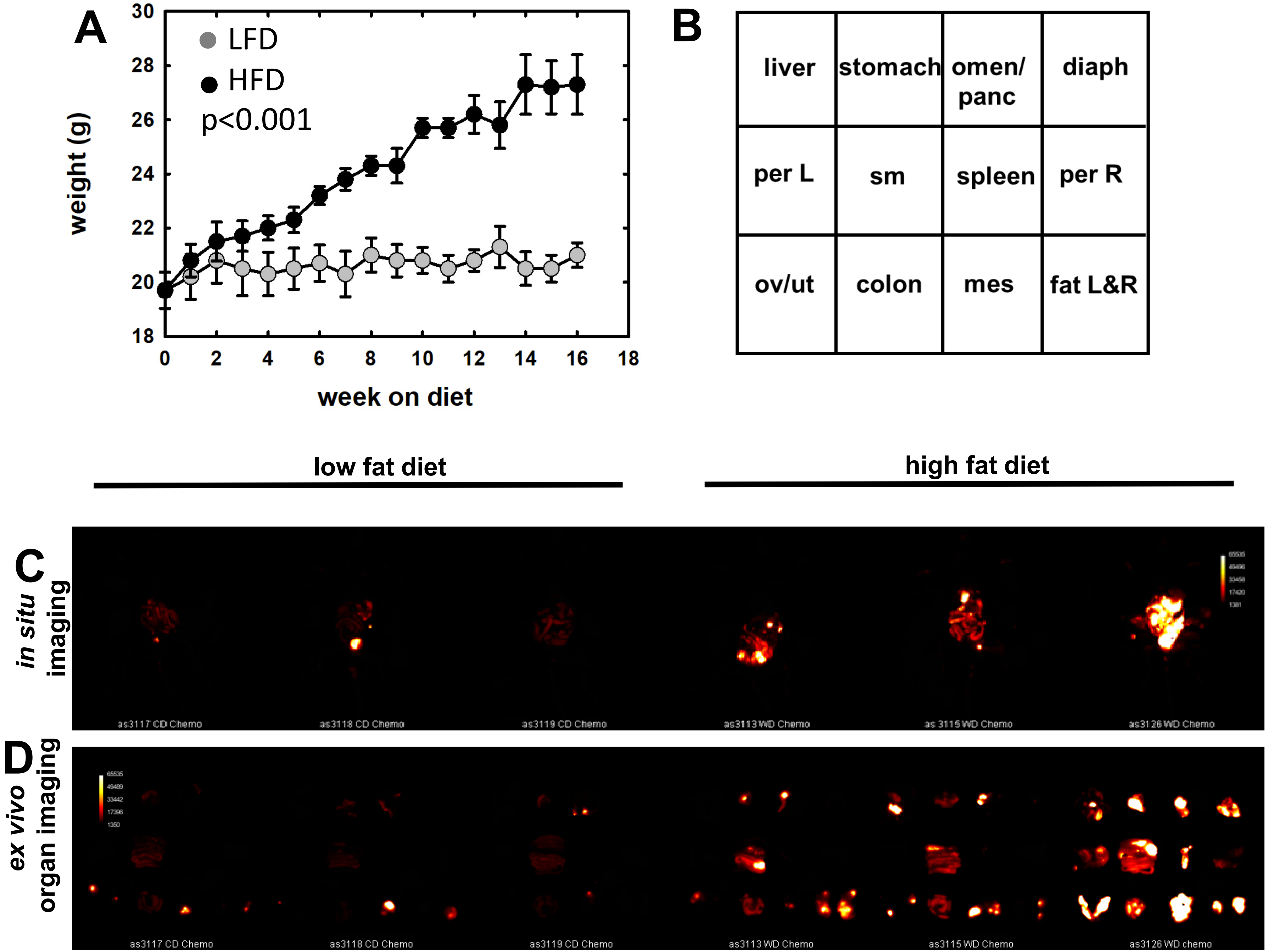

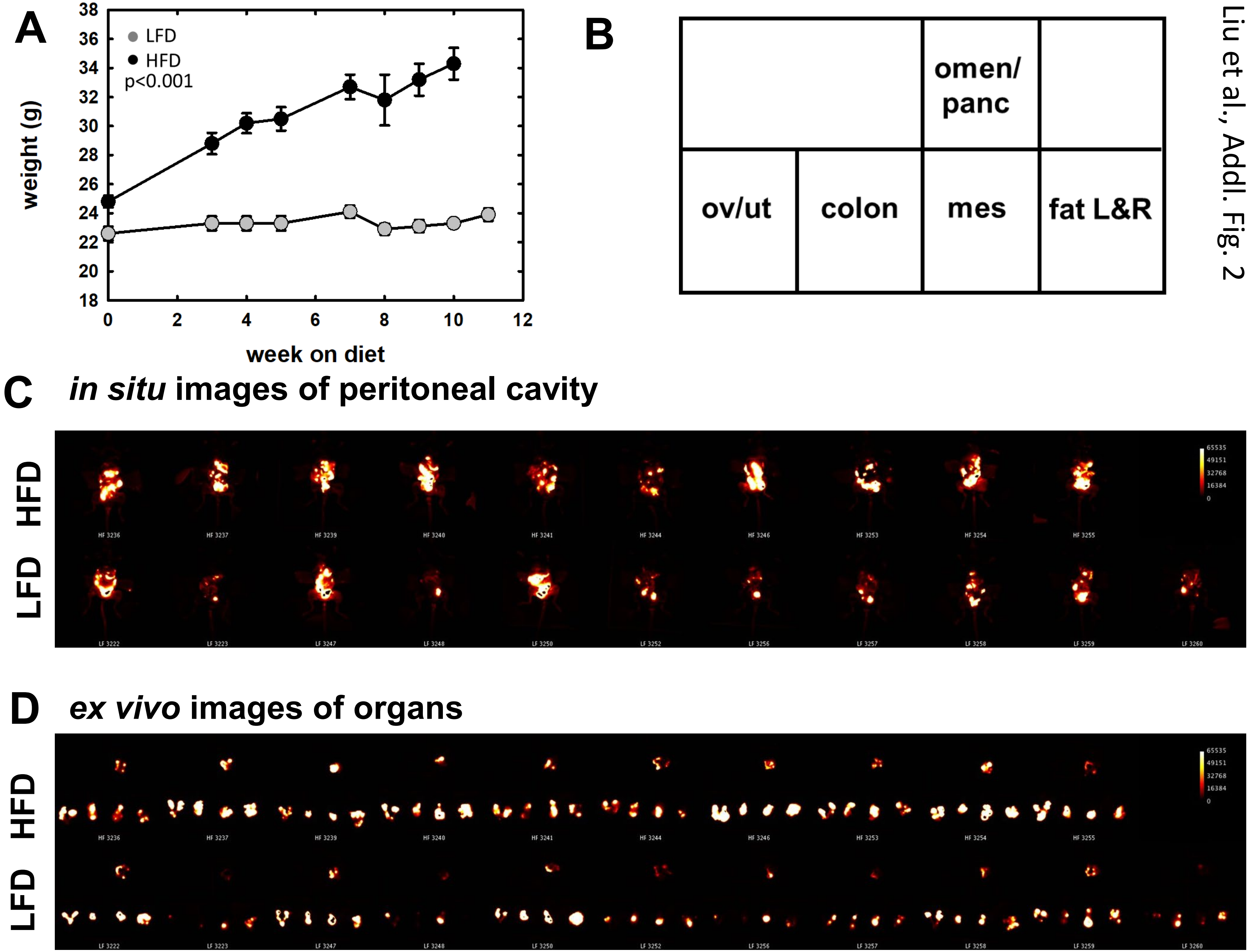

Liu et al., Addl. Fig. 2

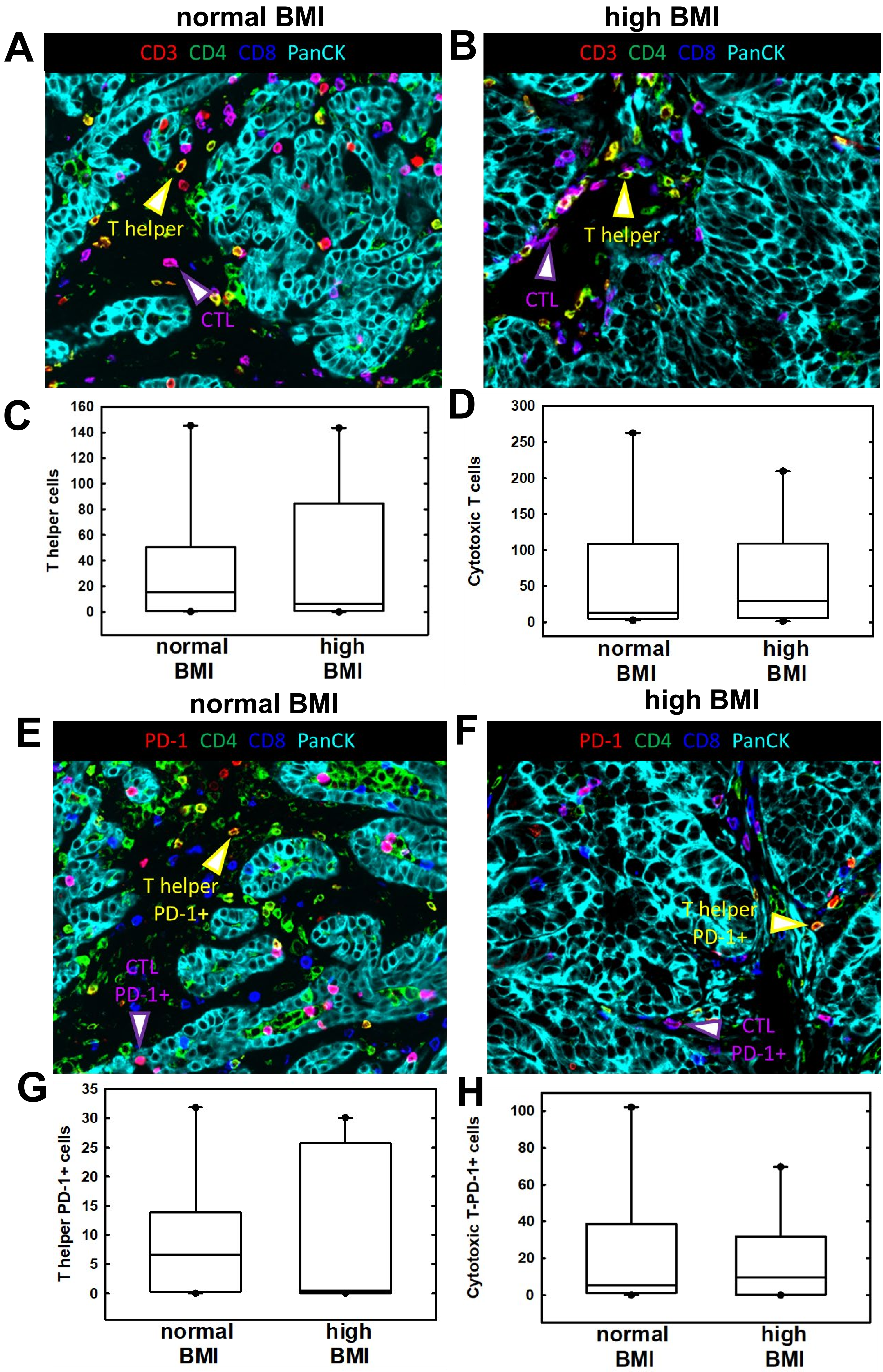
normal BMI
high BMI
A
B
CD3 CD4 CD8 PanCK
CD3 CD4 CD8 PanCK
T helper
CTL
T helper
CTL
C
D
T helper cells
Cytotoxic T cells
normal BMI
high BMI
normal BMI
high BMI
normal BMI
high BMI
E
F
PD-1 CD4 CD8 PanCK
PD-1 CD4 CD8 PanCK
T helper PD-1+
CTL PD-1+
T helper PD-1+
CTL PD-1+
G
H
T helper PD-1+ cells
Cytotoxic T-PD-1+ cells
normal BMI
high BMI
normal BMI
high BMI

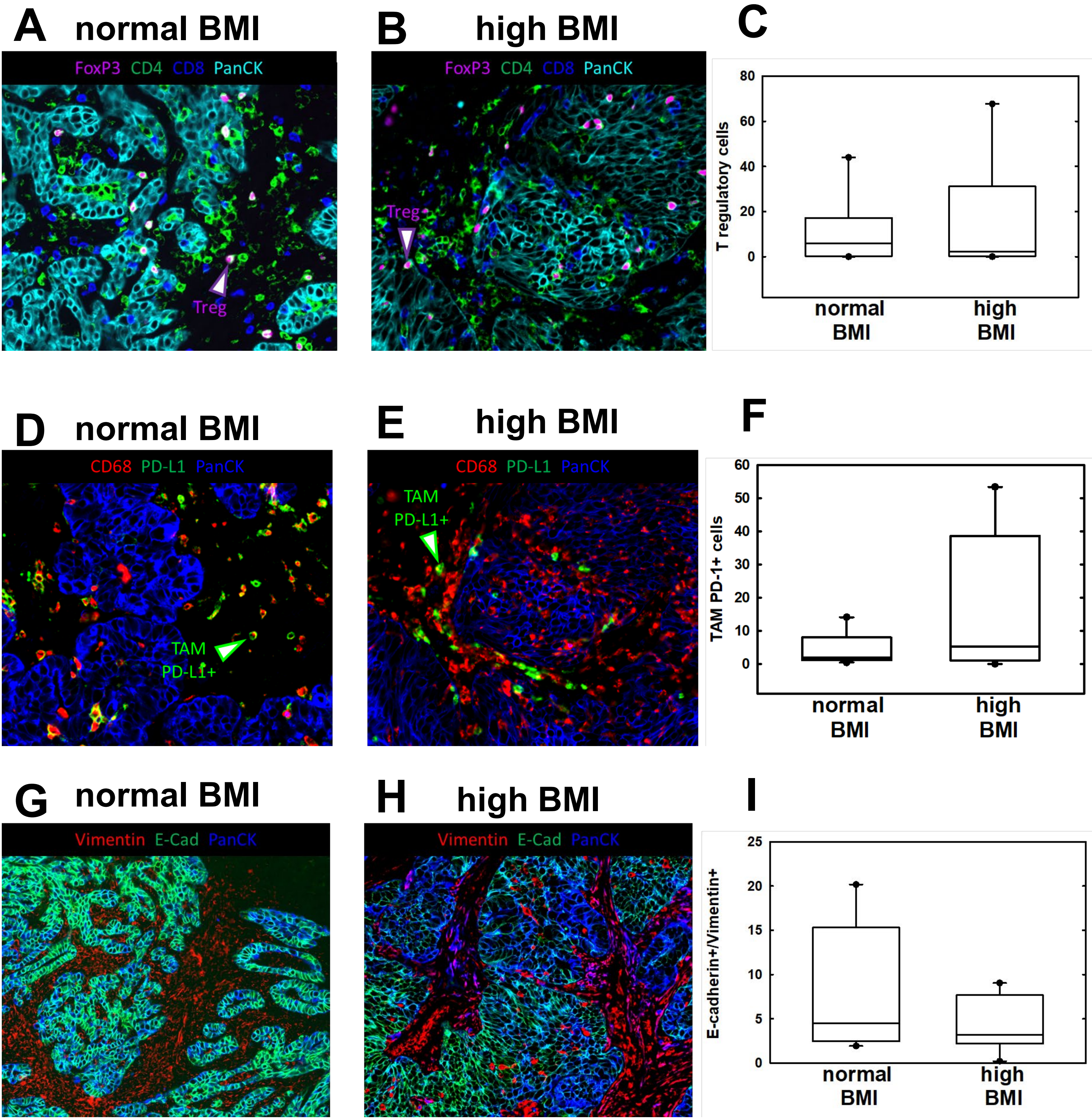

A normal BMI
FoxP3 CD4 CD8 PanCK
Treg
B high BMI
FoxP3 CD4 CD8 PanCK
Treg
C
T regulatory cells
80
60
40
20
0
normal BMI
high BMI
D normal BMI
CD68 PD-L1 PanCK
TAM PD-L1+
E high BMI
CD68 PD-L1 PanCK
TAM PD-L1+
F
TAM PD-1+ cells
60
50
40
30
20
10
0
normal BMI
high BMI
G normal BMI
Vimentin E-Cad PanCK
H high BMI
Vimentin E-Cad PanCK
I
E-cadherin+/Vimentin+
25
20
15
10
5
0
normal BMI
high BMI

Liu et al, Addl. Fig. 5

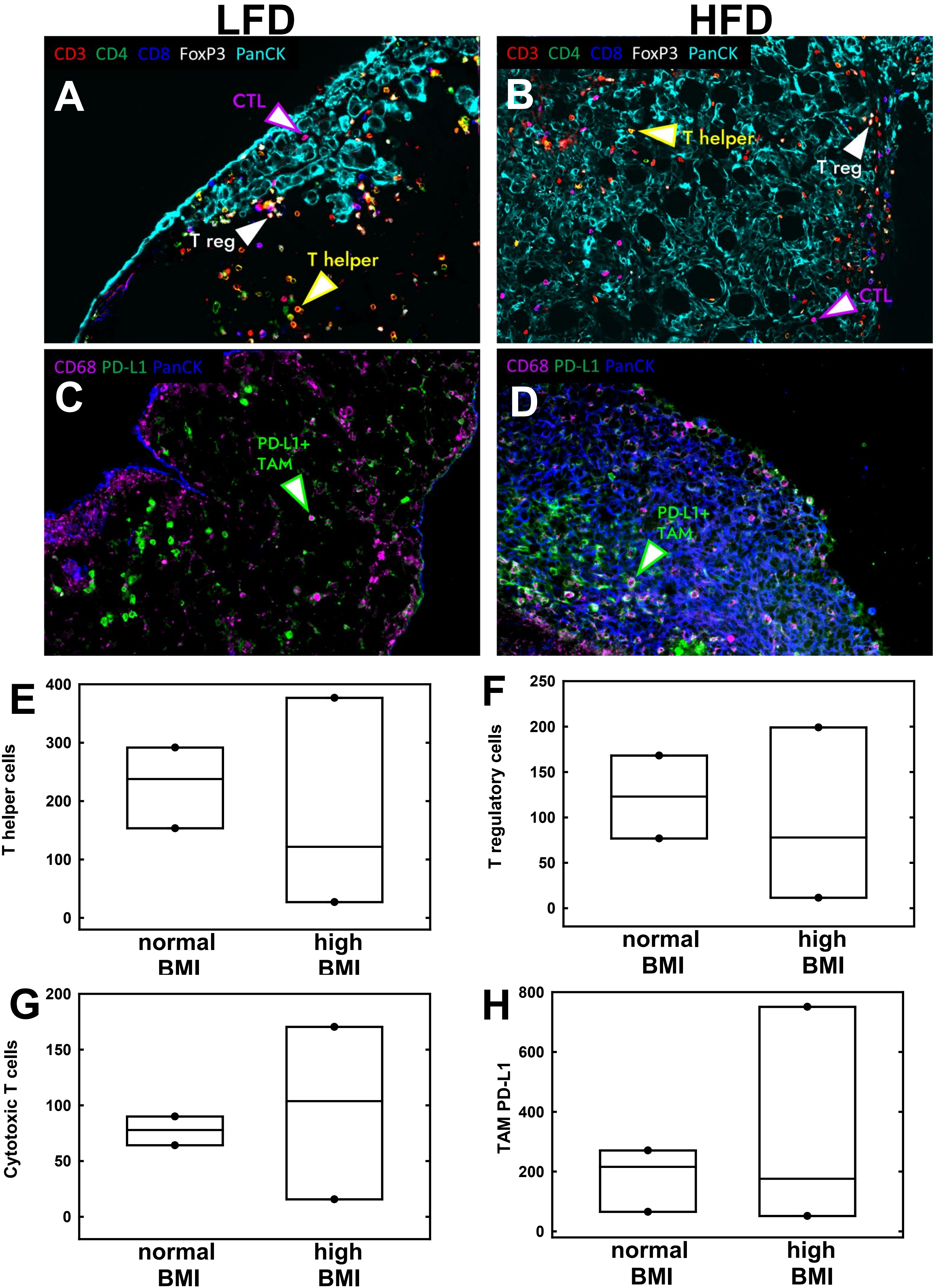

Supplement: Supplementary file 1 — Additional file 1: Additional methods. Additional Table 1. Phenotyping of murine immune cells. Additional Table 2. Additional adipokine array analysis of ascites from mice on low fat diet (LFD) or high fat diet (HFD). Additional Fig. 1. Pilot pre-clinical trial cohort of high fat diet (HFD) and chemotherapy response. Additional Fig. 2. Complete data from pre-clinical trial of HFD and chemotherapy response. Additional Fig. 3. Evaluation of immune cell staining in human ovarian tumors from patients with normal vs high body mass index (BMI). Additional Fig. 4. Additional immune cell and epithelial-mesenchymal transition (EMT) marker staining in human ovarian tumors from patients with normal vs high BMI. Additional Fig. 5. Evaluation of immune cell staining in murine tumors from mice on a control low fat diet (LFD) or high fat diet (HFD). [file 13046_2023_2740_MOESM1_ESM.pdf]
